# Management of Metastatic Solid Pseudopapillary Cancer of the Pancreas: A Case Report

**DOI:** 10.4021/wjon692w

**Published:** 2013-09-27

**Authors:** Matthew Dixon, John Cannon, Daniel Kagedan, Corwyn Rowsell, Laurent Milot, Yoo-Joung Ko, Natalie Coburn

**Affiliations:** a Department of Surgery, Maimonides Medical Center, Brooklyn, NY, USA; b Department of Biology, Carleton College, Northfield, MN, USA; c Department of Surgery, University of Toronto, Toronto, Ontario, Canada; d Department of Anatomical Pathology, University of Toronto, Toronto, Ontario, Canada; e Department of Radiology, University of Toronto, Toronto, Ontario, Canada; f Department of Medicine, University of Toronto, Toronto, Ontario, Canada

**Keywords:** Solid pseudopapillary tumor, Pancreas, Metastasis, Surgery

## Abstract

Solid pseudopapillary tumors (SPT) of the pancreas are rare neoplasms predominantly found in females. The tumors are often histologically benign and patient outcomes are correspondingly favorable. This report presents the case of a 21-year-old woman who presented with metachronous metastatic SPT, and details the diagnosis and management of this patient. The patient underwent a distal pancreatectomy for resection of the primary neoplasm with negative margins. A surveillance ultrasound performed at 43 months post-operatively revealed new hepatic lesions; these lesions were surgically resected and pathologically demonstrated to be metastatic SPT of the pancreas. This case report demonstrates the potential for latent metastasis of resected SPT, imaging characteristics of metastatic disease, the need for surveillance of patients following resection of SPT of the pancreas and a review of relevant literature on SPT.

## Introduction

Solid pseudopapillary tumors (SPT) of the pancreas were first described by Virginia Frantz in 1959 as a “papillary tumor of the pancreas, benign or malignant [1].” These tumors are often characterized by solid and cystic components, with cellular degenerative changes alternating with pseudopapillary formation [2, 3]. SPT are rare neoplasms, accounting for 1-2% of pancreatic cancers [4]. More than 90% of SPT are diagnosed in women during their second to fourth decade of life [5]. The majority of SPT are located in the pancreatic body and tail [6-8], with the pathogenesis of these tumors remaining largely undefined [5]. Aggressive surgical therapy has evolved as the mainstay of treatment [2, 4, 9]. Most commonly, SPT are histologically benign; however approximately 10% to 15% of SPT are found to harbor malignant features, which are often low grade [10]. The malignant potential of SPT is low [4, 5], with rare reports of metastatic spread. This case report describes the diagnosis and management of a patient with metachronous metastatic SPT.

## Case Report

**Figure 1:**
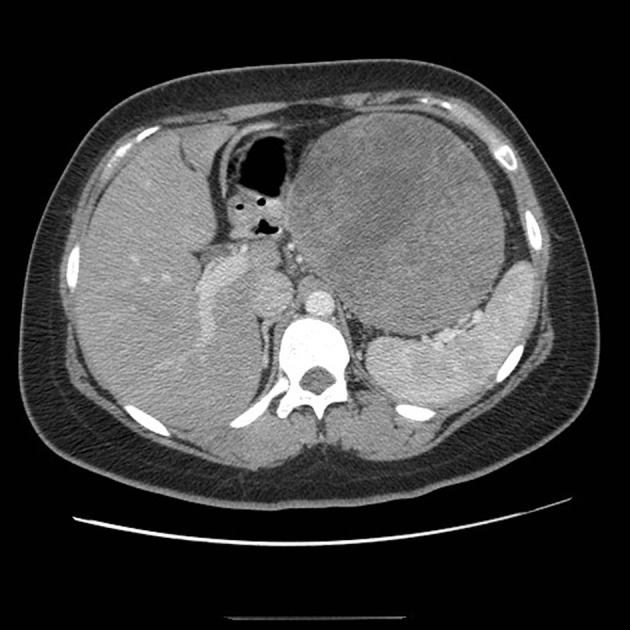
CT scan of the abdomen demonstrating a large mass arising from the tail of the pancreas.

**Figure 2:**
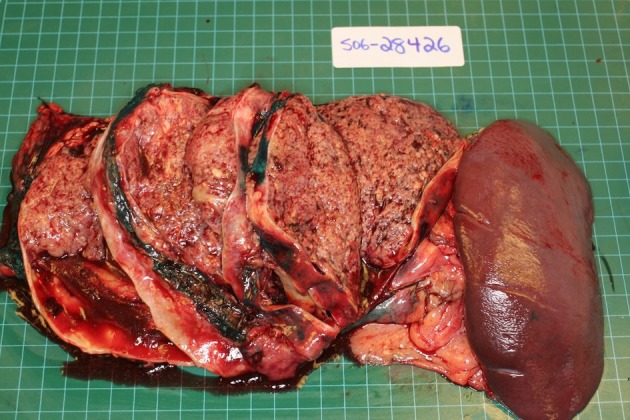
Surgical specimen demonstrating large tumor in the tail of the pancreas with prominent cystic degeneration.

**Figure 3:**
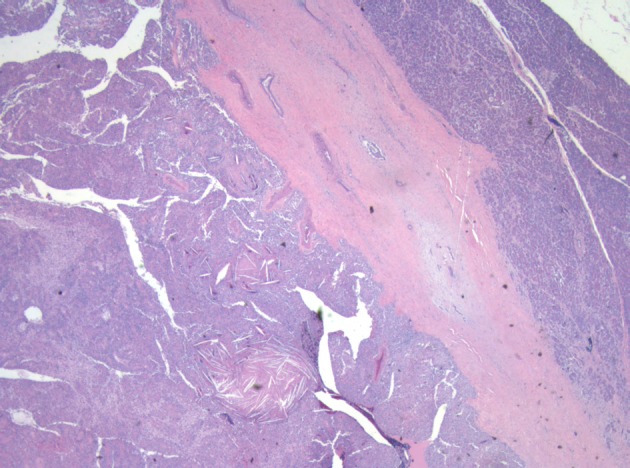
Low power micrograph showing normal pancreas (right) and tumor (left). Note cholesterol clefts in tumor.

A 21-year-old female patient presented with vague left upper quadrant pain. She did not smoke or consume alcohol, and her only medication was an oral contraceptive. She appeared well, with no abnormal physical exam findings. Routine laboratory studies including liver function tests were all within normal limits. An abdominal ultrasound (AUS) revealed a large mass which appeared to originate from the tail of the pancreas. Computerized tomography-(CT)-scan demonstrated a 12 cm mass arising from the tail of the pancreas and no evidence of metastases (Fig. 1). The patient was taken to the operating room where an exploratory laparotomy confirmed that the large mass arose from the tail of the pancreas. There was no evidence of peritoneal or other distant metastases. The tumor was found to be adherent to the posterior wall of the stomach as well as to the greater omentum. A distal pancreatectomy with en-bloc splenectomy, partial gastrectomy and omentectomy was performed (Fig. 2). Pathology revealed a solid pseudopapillary tumor of the pancreas (Fig. 3), with negative resection margins. Thirteen lymph nodes were examined with the specimen, all negative for metastases. The tumor cells were positive for CD10 and alpha 1-antitrypsin, and focally positive for synaptophysin. The tumor was negative for neuron-specific enolase and chromogranin. The patient’s immediate post-operative course was complicated by pneumonia treated with oral antibiotics. She received appropriate post-splenectomy vaccinations, and was discharged home on post-operative day (POD) 7.

**Figure 4:**
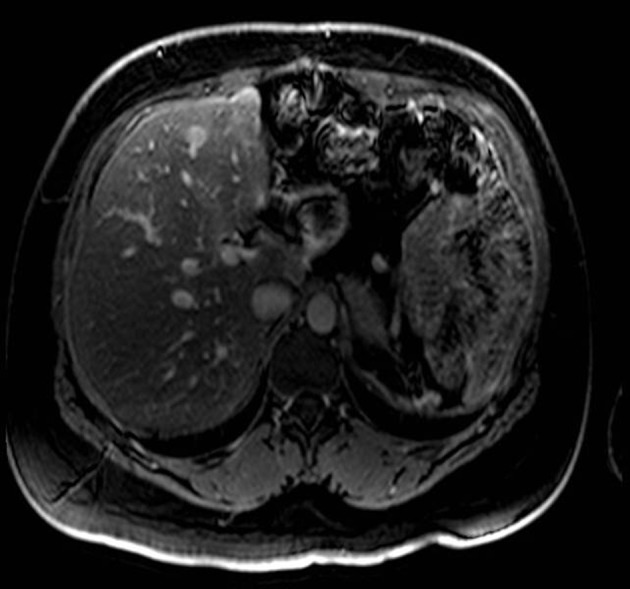
MRI with gadolinium contrast demonstrating a metastases in segment 4 of the liver.

**Figure 5:**
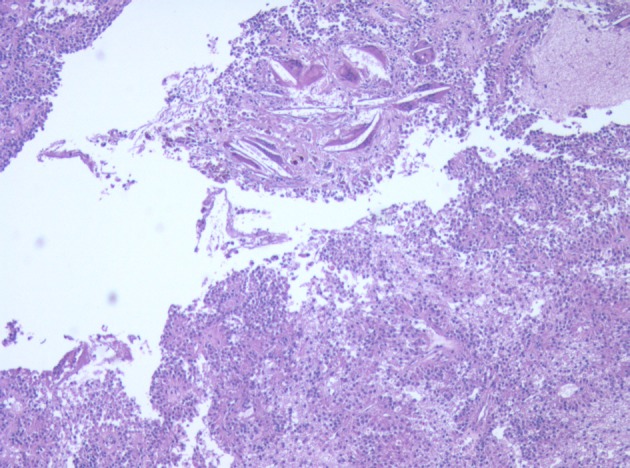
Micrograph of one of the liver metastases. The tumor demonstrates neoplastic cells supported by delicate vessels, pseudopapillary growth pattern, and cholesterol clefts.

Surveillance AUS was performed biannually, then annually. A surveillance AUS performed 43 months post-operatively demonstrated fatty transformation of the liver with a discrete hypoechoic lesion measuring 1.1 × 1.0 × 0.8 cm in segment 4B/5 of the liver. There was no evidence of local recurrence. The patient was asymptomatic, with no focal findings on physical exam. Laboratory studies including liver function tests were within normal limits. Magnetic Resonance Imaging (MRI) demonstrated a 1-cm lesion in segment 4B, and a 0.8 cm lesion in segment 5 of the liver (Fig. 4). Both lesions demonstrated intermediate high signal on T2 weighted images and progressive enhancement after gadolinium injection. They demonstrated restricted diffusion on diffusion weighted imaging. They were suspicious for metastases, but atypical hemangiomas remained in the differential. In order to narrow this differential, a contrast enhanced ultrasound study was performed, and the lesions demonstrated mild arterial enhancement with rapid complete washout in keeping with metastases. These lesions were not avid on Fluorodeoxyglucose-Positron Emission Tomography (FDG-PET). The patient underwent for a diagnostic laparoscopy and US-guided biopsy of her hepatic lesions. A definitive diagnosis could not be made intra-operatively based on intra-operative frozen section analysis of the needle biopsy, however the pathologist favored malignancy rather than a benign etiology. Therefore, the procedure was converted to a laparotomy with resection of segments 4b and 5, as well as a cholecystectomy. An enlarged periportal lymph node was excised as well. Pathology revealed 2 foci of metastatic solid pseudopapillary tumor of the pancreas in the liver (Fig. 5), measuring 8 mm and 7 mm. The enlarged lymph node was negative for malignancy. She recovered uneventfully from this procedure and was discharged home on POD 5.

A re-staging CT scan of her abdomen and pelvis was obtained one month post-operatively and demonstrated no evidence of intra-abdominal disease. However, an MRI performed concurrently demonstrated two lesions: a 4 mm lesion in segment 6, and a 7 mm lesion in segment 7. In retrospect, these lesions were visible on prior MRI, and were unchanged. After discussion at a multi-disciplinary hepato-pancreatico-biliary tumour board and review of the literature, she underwent 6 cycles of adjuvant gemcitabine chemotherapy at a dose of 1,000 mg/m^2^ (weekly × 3 every 4 weeks) for 6 months. She tolerated the chemotherapy without significant side effects. She remains without recurrence of disease 41 months post-hepatic resection. She continues follow-up with MR imaging of the abdomen every 6 months.

## Discussion

Solid pseudopapillary tumors of the pancreas are a rare primary neoplasm and are estimated to constitute 1-2% of all pancreatic tumors and 13% of all resected cystic neoplasms of the pancreas [11]. SPT are found predominantly in females with an approximate female-predominance of 10:1. The majority of SPT affects patients between the 2nd and 4th decade of life [12]. SPT are usually benign, and malignant features are estimated to be present in only 10-15% of cases [10].

The cellular origins of SPT are unclear, however reports have suggested these tumors have ductal epithelial, neuroendocrine, multi-potent primordial cell, and possibly extra-pancreatic genital ridge angle-related cell origins [6]. The predominance of SPT in female patients and presence of progesterone receptors in 80% of these tumors raises the possibility that SPT growth may be influenced by sex hormones [13] SPT most often present as well-circumscribed tumors with a combination of solid and cystic components along with cellular degenerative changes alternating with pseudopapillary formation [2, 3]. These histological features are useful in distinguishing solid pseudopapillary tumors from other neoplasms [2, 3].

SPT often present with abdominal or back pain due to tumor mass effect; a palpable mass may also be present [11]. Pre-operative diagnosis is challenging due to difficulties differentiating this tumor from other neoplasms of the pancreas, however the use of MRI may improve diagnostic accuracy [14]. Although there has been an increase in recognition of SPT, the majority of data on these tumors comes from case reports and few large cohort studies exist.

Surgical resection is the recommended treatment for SPT with both benign and malignant features [5]. Patient outcomes following complete resection are excellent with 5-year survival rates of up to 97% [2, 3]. In cases of metastatic SPT, surgical metastasectomy is the preferred method of treatment. The use of adjuvant chemotherapy or radiotherapy in the treatment of SPT is limited to isolated case studies [15].

This report presents a noteworthy case of SPT due to the rarity of metastatic SPT, and the prolonged disease-free interval prior to the detection of hepatic metastases. Furthermore, the metastases occurred despite achievement of microscopically tumor-free surgical margins in the removal of the primary neoplasm. Previous reports of metastasis following resection of SPT have generally occurred in cases in which microscopically or grossly positive margins were obtained [8]. Given the young age of the patient and the R0 resection, we opted to treat the patient with gemcitabine.

While the majority of SPT have a relatively indolent disease trajectory, this does not preclude the potential for metastatic progression. Due to the limited data available on SPT and the relative rarity of aggressive behavior, it is difficult to identify, either clinically or pathologically, those cases of SPT with metastatic potential. Furthermore, as demonstrated by this case, metastases may develop even following surgical resection of the primary neoplasm with negative margins. Consequently, physicians caring for these patients should maintain an index of suspicion for metastatic progression, including development of metachronous lesions, and must be prudent to closely follow these patients.

Due to the rarity of SPT, evidence-based guidelines regarding post-operative follow-up do not exist. Biannual physical examinations combined with annual CT scans of the thorax and abdomen should be considered. Given that SPT is most often diagnosed in young females, AUS may be a preferable surveillance methodology. As is demonstrated by this case and elsewhere in the literature, there can be a significant disease-free interval between resection of the primary tumor and the development of metastases. Therefore, annual imaging studies may be performed for a minimum of 10 years following definitive tumor resection.
